# Radiosurgery in Grade II and III Meningiomas: A Systematic Review and Meta-Analysis

**DOI:** 10.3390/jpm14080802

**Published:** 2024-07-29

**Authors:** Amin Jahanbakhshi, Masoumeh Najafi, Marzieh Gomar, Patrizia Ciammella, Maria Paola Ruggieri, Cinzia Iotti, Sebastiano Finocchi Ghersi, Anne-Agathe Serre, Lilia Bardoscia, Angela Sardaro, Sophie Boisbouvier, Camille Roukoz, Salvatore Cozzi

**Affiliations:** 1 Skull Base Research Center, Rasool Akram Hospital, Iran University of Medical Sciences, Tehran 1997667665, Iran; jahanbakhshi.a@iums.ac.ir (A.J.); najafi.mas@iums.ac.ir (M.N.); 2 Radiation Oncology Research Center, Iran Cancer Institute, Tehran University of Medical Sciences, Tehran 1997667665, Iran; m.gomar1365@gmail.com; 3 Radiation Oncology Unit, Azienda USL-IRCCS di Reggio Emilia, 42123 Reggio Emilia, Italy; patrizia.ciammella@ausl.re.it (P.C.); mariapaola.ruggieri@ausl.re.it (M.P.R.); cinzia.iotti@ausl.re.it (C.I.); sebastiano.finocchighersi@gmail.com (S.F.G.); 4 Radiation Oncology Department, Centre Leon Berard, 69373 Lyon, France; anne-agathe.serre@lyon.unicancer.fr (A.-A.S.); sophie.boisbouvier@lyon.unicancer.fr (S.B.); 5 Radiation Oncology Unit, S. Luca Hospital, Healthcare Company Tuscany Nord Ovest, 55100 Lucca, Italy; lilia.bardoscia@uslnordovest.toscana.it; 6 Interdisciplinary Department of Medicine, Section of Radiology and Radiation Oncology, University of Bari “Aldo Moro”, 70124 Bari, Italy; angela.sardaro@uniba.it

**Keywords:** meningiomas, SBRT, literature review, grade II and III meningiomas, radiotherapy

## Abstract

Background: Meningiomas are the most prevalent benign intracranial tumors. When they are of the invasive subtypes, i.e., grades II and III, they can recur rapidly and present a real challenge for physicians. This study is focused on the use of stereotactic radiosurgery to manage high-grade meningiomas. Method: Medline via PubMed was searched from inception to December 2022 to retrieve studies on stereotactic radiation therapy for patients with grade II-III meningiomas. This study was conducted under PRISMA guidelines. Result: A total of 29 articles involving 1446 patients with grade II-III meningiomas treated with stereotactic radiation therapy were included in the present study. Of these studies, 11 were conducted exclusively on patients with atypical meningiomas (grade II), 1 targeted anaplastic meningiomas (grade III), and 17 articles were carried out on both grade II and III meningiomas. The pooled 1, 2, 3, 5, and 10-year overall survival (OS) of grade II meningiomas was 0.96 [*p* < 0.01], 0.89 [*p* = 0.01], 0.90 [*p* = 0.09], 0.81 [*p* < 0.01], and 0.66 [*p* = 0.55], respectively. The pooled 2, 5, and 10-year OS of grade III meningiomas was 0.64 [*p* = 0.01], 0.41 [*p* = 0.01], and 0.19 [*p* < 0.01], respectively. Conclusions: Although long-term prospective studies are still required, the outcomes of stereotactic radiation therapy appear promising regarding overall outcome and progression-free survival.

## 1. Introduction

Meningiomas are the most common benign primary central nervous system (CNS) tumor [1,2,3,4]. The majority of diagnosed meningiomas are WHO grade I tumors. In 10% of cases, meningiomas show signs of malignancy with increased recurrence rates (30–50% for WHO grade II and 50–94% for WHO grade III) and a worse prognosis [5,6,7]. Males are more likely to have aggressive meningiomas [8]. Patients with WHO grade II-III meningiomas have a shorter OS and higher recurrence rates. Anaplastic meningiomas (grade III) have a mean overall survival (OS) of 2 years despite receiving the best possible surgical and adjuvant care. It is shown that grade II meningiomas are around 8 times more likely to recur than benign meningiomas at the same age and sex. Moreover, recurring meningiomas typically exhibit greater aggressiveness than the initial tumor [7]. This is why in clinical practice, while being very uncommon, WHO grade II and III meningiomas are considered aggressive and are far more challenging to cure than benign meningiomas [6]. Malignant lesions may develop from lower-grade meningiomas over 2 to 16 years, with a risk estimate of 0.04 to 2.6% at 15 years [9,10,11,12].

When surgery is not an option, radiation therapy (RT) has become a viable alternative therapeutic option to limit tumor development. It has also been demonstrated that using stereotactic radiosurgery (SRS) as an adjuvant tool enhances progression-free survival (PFS) and local control (LC) in all meningioma grades [13,14,15]. 

In recent years, advances in RT planning and delivery techniques, in particular the advent of the new-generation linear accelerator (LINAC) with the flattening filter-free (FFF) mode, have improved treatment accuracy and given rise to the adoption of ultra-hypofractionated RT schedules in the form of SRS or stereotactic body radiotherapy (SBRT) in different oncological settings, with an acceptable toxicity profile [16,17,18,19,20,21,22,23,24,25].

SRS and fractionated stereotactic radiotherapy (FSRT) are examples of RT techniques. SRS uses stereotactic image guidance to deliver radiation with a high dose in a single fraction, but FSRT uses smaller doses delivered over three to five fractions. 

Meningiomas have been observed to respond well to RT, with an increase in PFS over the long run [26,27]. Normofractionated external beam radiotherapy (EBRT) is still an option when SRS is not appropriate, such as when the tumor is large, rapidly growing, and causing noticeable symptoms, or when it is close to or involves critical structures like the cavernous sinus or the optic pathway. Numerous studies have shown that FSRT, either as the initial treatment for unresectable meningiomas such as optic nerve sheath and cavernous sinus meningiomas or after partial tumor resection, reduces tumor recurrence and improves OS [28,29]. Comparing surgical series, some studies have shown a similar tumor control rate with a lower complication rate in SRS treatment [30,31]. 

Many radiosurgical series lack data regarding the histopathologic subtypes and there is little information available about the results of SRS for patients with aggressive meningiomas. This study aims to systematically review the clinical outcomes of SRS in the management of WHO grade II and III meningiomas.

## 2. Materials and Methods

### 2.1. Object

This study aimed to investigate the clinical efficacy of SRS in managing patients with WHO grade II and III meningiomas. This study is designed following the Preferred Reporting Items for Systematic Reviews and Meta-Analyses (PRISMA) guidelines and the registration Prospero ID is 5518835 [32].

### 2.2. Search Strategy 

To retrieve studies addressing the clinical efficacy of SRS for high-grade meningiomas, Medline via PubMed was searched from inception to December 2022. No restrictions were made on the type of studies except for case reports. The gray literature was also reviewed through a manual search of Google Scholar. The relevant keywords were obtained from reviewing the literature and the electronic databases were searched using “Meningioma”, “stereotactic radiosurgery”, “stereotactic radiotherapy”, and “radiation treatment”, with an appropriate Boolean operator of AND/OR/NOT.

### 2.3. Eligibility Criteria

Studies meeting both the inclusion and exclusion criteria were considered for data extraction. The inclusion and exclusion criteria are as follows:

Inclusion criteria:
  1. English articles.
  2. Studies on humans.
  3. Patients diagnosed with WHO grade II and III primary or recurrent meningiomas.
  4. Original articles.
Exclusion criteria:
  1. Non-English articles.
  2. In vivo or in vitro studies.
  3. Patients with grade I meningiomas.
  4. Review articles, conference abstracts, letters to editors, book chapters, and case reports.
  5. Articles in which outcome data were missing.

### 2.4. Study Selection

Two reviewers independently conducted the study selection process in two steps, a title/abstract assessment and a full-text assessment. The retrieved studies from the search of electronic databases were considered for the study selection process. First, the bi-step title/abstract process was carried out and the articles relevant to the present study object were considered for full-text assessment. Second, in the full-text assessment process, the articles fully fitting our eligibility criteria were considered for the data extraction process. 

### 2.5. Data Extraction

Two reviewers independently extracted the data from the final included articles. The following information of the articles was extracted: (1) first author name, year of publication, age, gender, type of study, primary or recurrent lesion, WHO grade of meningioma, and occurrence of surgery; (2) RT modality, RT dose, number of fractions, LC, and survival outcomes. The extracted data were checked by a third reviewer.

### 2.6. Data Synthesis 

The percentages of OS and PFS were used as effect sizes at each time point of the 1, 2, 3, 4, 5, and 10-year OS and the 1, 2, 3, 5, and 10-year PFS. The standard error (SE) of the observed proportion was first calculated as well as the proportion of OS and PFS at each time point with their SE. The I^2^ statistic was applied to quantify the heterogeneity of the studies and an I^2^ level of 0–40% was regarded as not important heterogeneity, 30–60% as moderate heterogeneity, 50–90% as substantial heterogeneity, and 75–100% as considerable heterogeneity. The pooled proportion of OS and PFS was calculated by using a fixed-effects model when the heterogeneity of the cumulative proportion was I^2^ < 40%, and if not, a random-effects model was used. The calculated pooled proportion of OS and PFS at each time point was represented with their 95% confidence interval. SATA version 17 software was used for the meta-analysis of the included studies.

## 3. Results

### 3.1. Study Characteristics 

**Figure 1:**
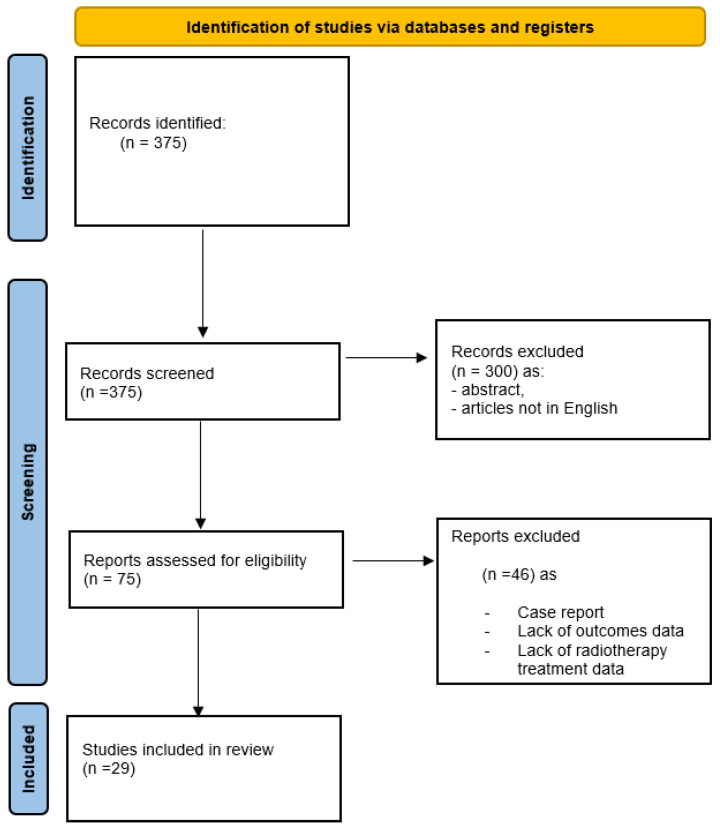
The PRISMA flowchart.

A total of 375 articles were obtained from the electronic databases. In the title/abstract screening step, 300 articles were removed from the selection process (abstract, review of the literature, articles not in English) and 75 articles were used in the full-text assessment process. A total of 29 articles involving 1446 patients diagnosed with atypical or anaplastic meningiomas were finally included for quantitative data synthesis. The PRISMA flowchart demonstrates the study selection process (Figure 1). Table 1 and Table 2 summarize the studies that examined the impact of SRS treatment on WHO grade II and III meningiomas (Table 1 and Table 2).

### 3.2. Patient and Treatment Characteristics

A total of 1446 patients were included. WHO grade II meningiomas were diagnosed in 1121 patients, while 277 patients had WHO grade III meningiomas. Forty-eight patients had either WHO grade II or III meningiomas. Age and sex were not specified depending on the WHO grade in every study. The median follow-up, when specified, was 41.5 months. Gamma knife SRS was used for 1249 patients, LINAC SRS for 72 patients, and Cyberknife SRS for 113 patients, and 12 patients received either LINAC or Cyberknife SRS. When specified, the average dose was 15.9 Gy.

### 3.3. PFS of WHO Grade II Meningiomas

**Figure 2:**
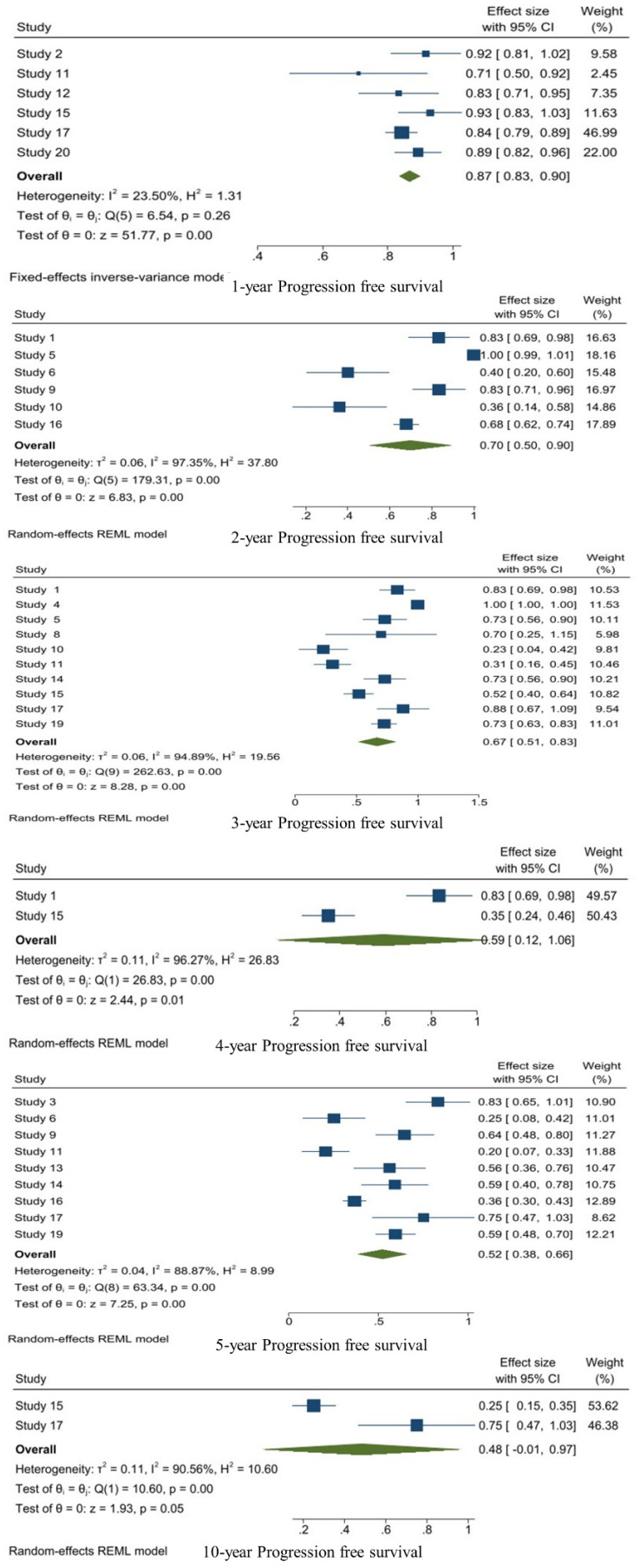
1-, 2-, 3-, 5-, and 10-year PFS analysis of WHO grade II meningiomas.

The fixed-effects model was used for 1-year PFS and others were calculated with a random-effects model. Six studies were included in the 1-year PFS analysis and the pooled 1-year PFS was 0.87 [95%CI: 0.83–0.90, I^2^: 23.5%, *p* = 0.26]. Also, six studies reported the 2-year PFS and the pooled 2-year PFS was 0.70 [95%CI: 0.50–0.90, I^2^: 97.35%, *p* < 0.01]. A total of 10 studies reported the 3-year PFS and it was 0.67 [95%CI: 0.51–0.83, I^2^: 94.89%, *p* < 0.01]. Two studies reported the 4-year PFS and the pooled 4-year PFS was 0.59 [95%CI: 0.12–1.06, I^2^: 96.27%, *p* < 0.01]. Nine studies described the 5-year PFS and it was 0.52 [95%CI: 0.38–066, I^2^: 88.87%, *p* < 0.01]. Two studies reported the 10-year PFS. The pooled 10-year PFS was 0.48 [95%CI: −0.01–0.97, I^2^: 90.56%, *p* < 0.01] (Figure 2).

### 3.4. OS of WHO Grade II Meningiomas

**Figure 3:**
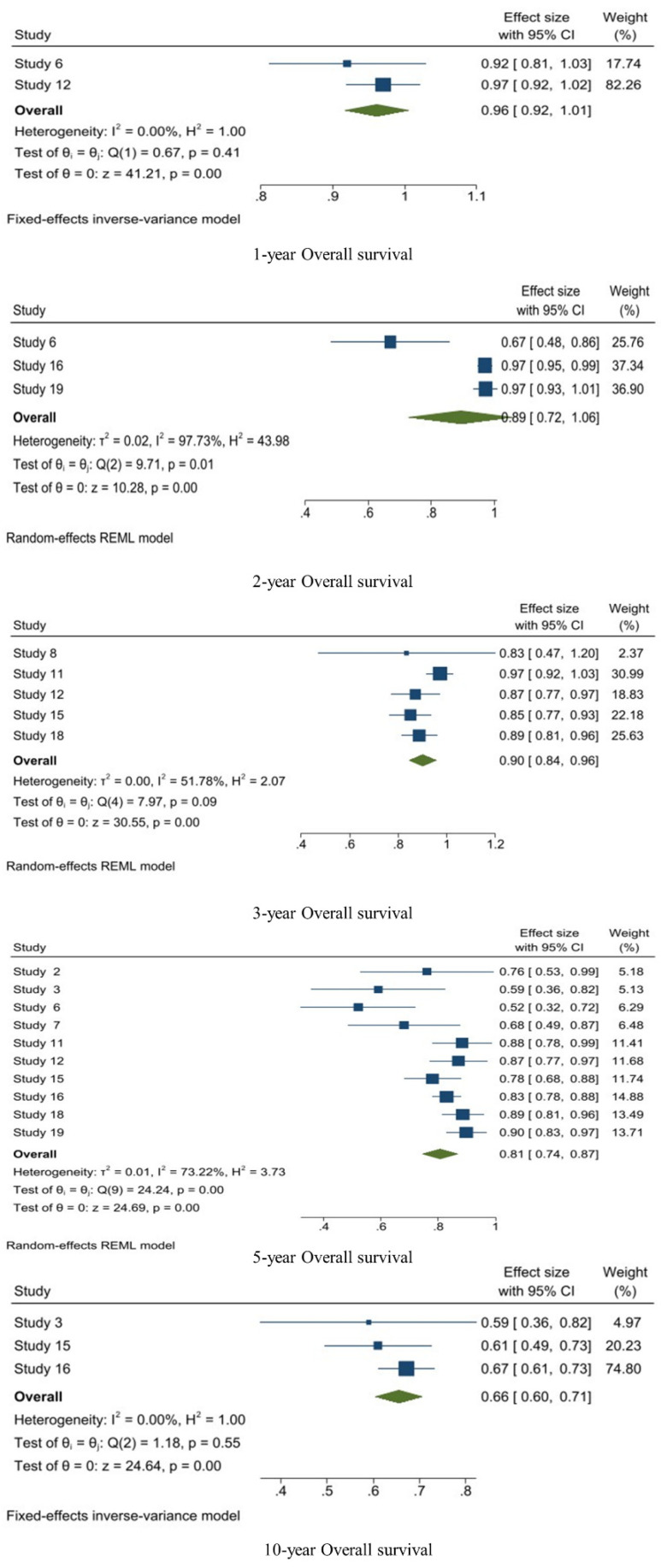
1-, 2-, 3-, 5-, and 10-year OS analysis of WHO grade II meningiomas.

A fixed-effects inverse-variance model was used for calculating the 1-year OS. The pooled 1-year OS was 0.96 [95%CI: 0.92–1.01, I^2^: 0.00%, *p* < 0.01]. Regarding the 2-year OS, the random-effects REML model was used and the pooled proportion of the 2-year OS was 0.89 [95%CI: 0.72–1.06, I^2^: 97.73%, *p* < 0.01]. A random-effects model was used for the 3-year OS and the pooled 3-year OS was 0.90 [95%CI: 0.84–0.96, I^2^: 51.78%, *p* = 0.09]. The pooled 5-year OS was calculated with random effects and it was 0.81 [95%CI: 0.74–0.87, I^2^: 73.22%, *p* < 0.01]. A fixed-effects model was applied for the 10-year OS and it was 0.66 [95%CI: 0.60–0.71, I^2^: 0.00, *p* = 0.55] (Figure 3).

### 3.5. PFS of WHO Grade III Meningiomas

**Figure 4:**
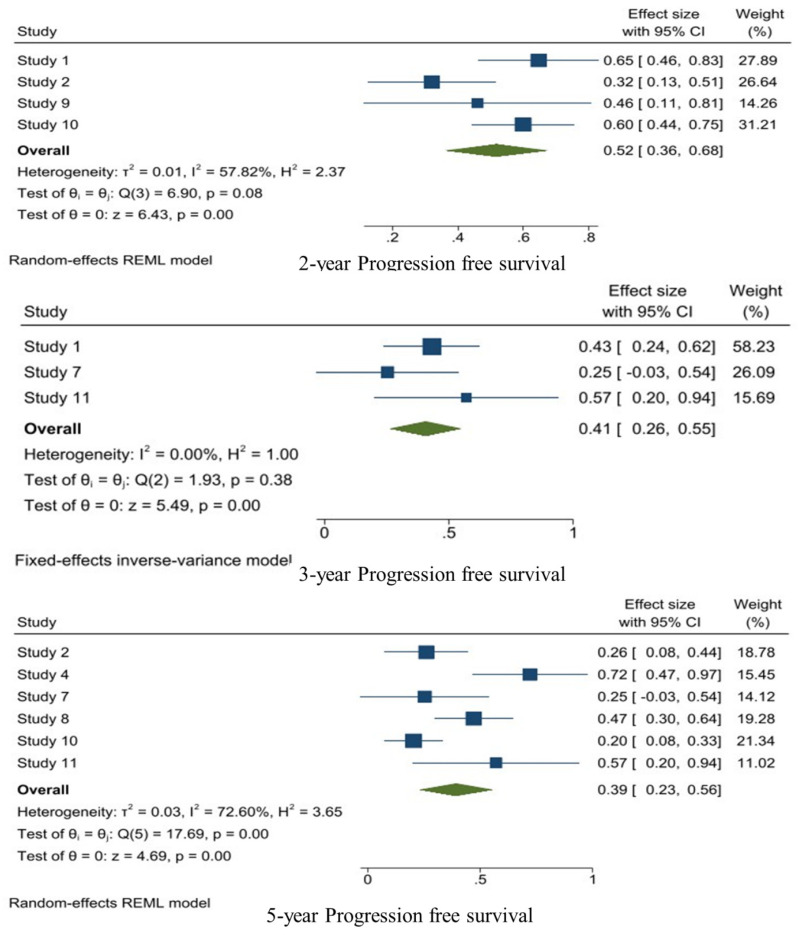
1-, 2-, 3-, 5-, and 10-year PFS analysis of WHO grade III meningiomas.

The random-effects model was used for the 2-year (I^2^: 57.82%) and 5-year PFS (I^2^: 72.60%) and the fixed-effects model was used for the 3-year PFS (I^2^: 0.0%). The 2-year PFS was reported in four studies and the pooled 2-year PFS was 0.52 [95%CI: 0.36–0.68, I^2^: 57.82%, *p* = 0.08). Three studies reported the 3-year PFS and the pooled 3-year PFS was 0.41 [95%CI: 0.26–0.55, I^2^: 0.0%, *p* = 0.38]. The 5-year PFS was reported in six studies and the pooled calculated 5-year PFS was 0.39 [95%CI: 0.23–0.56, I^2^: 72.60%, *p* = 0.00] (Figure 4).

### 3.6. Overall Survival of WHO Grade III Meningiomas

**Figure 5:**
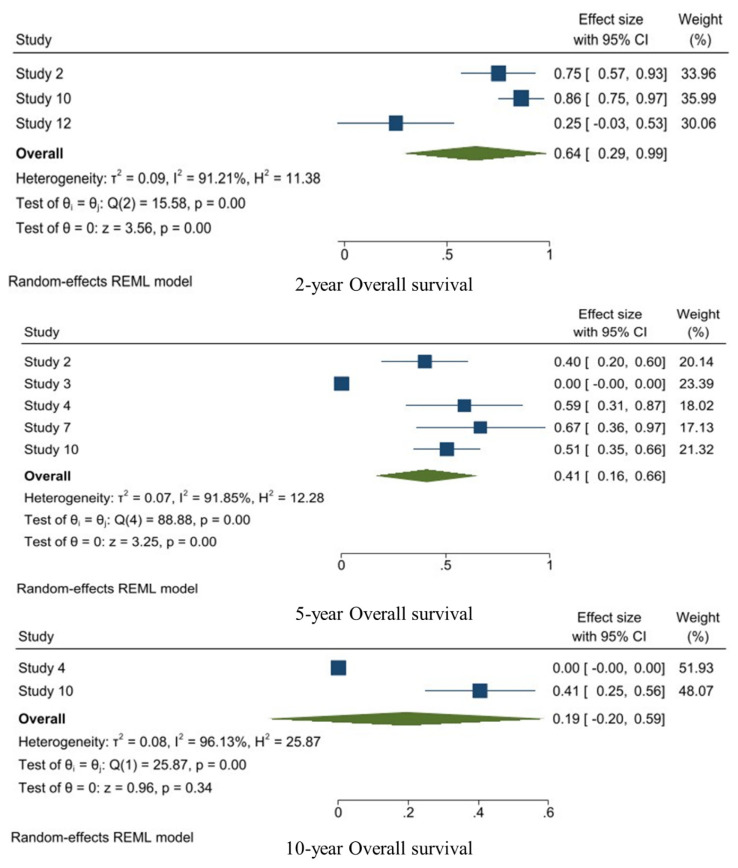
1-, 2-, 3-, 5-, and 10-year OS analysis of WHO grade III meningiomas.

The random-effects model was used for the 2-year, 5-year, and 10-year OS. The 2-year OS was reported in three studies and the pooled 2-year OS was 0.64 [95%CI: 0.29–0.99, I^2^: 91.85%, *p* < 0.01]. Moreover, five studies reported the 5-year OS and the pooled 5-year OS was 0.41 [95%CI: 0.16–0.66, I^2^: 91.85%, *p* < 0.01]. Two studies reported the 10-year OS, with a pooled 10-year OS of 0.19 [95%CI: −0.20–0.59, I^2^: 96.13%, *p* < 0.01] (Figure 5).

## 4. Discussion

Adjuvant RT following surgery of atypical or anaplastic meningiomas (WHO grade II-III) is a well-known treatment and has been used by many neurooncologists [62,63,64]. However, using SRS for these tumors either as an exclusive or adjuvant treatment is a new subject that is addressed rarely in the literature. This systematic review aimed to study the efficacity and outcomes of SRS in treating WHO grade II-III meningiomas.

For patients who cannot undergo major surgery or are hesitant to take on the upfront risks of open resection, SRS may be regarded as an alternative treatment option. SRS is often considered a second treatment option for atypical or malignant meningiomas if conventional RT and/or surgical excision have failed [65]. WHO grade II and III meningiomas are uncommon and predicting their response to treatment and their recurrence rates might be challenging. Choosing the most suitable therapy is always challenging. Indeed, to identify tumors that are linked to a higher recurrence rate and lower survival, more specific and reliable prognostic variables are required [66]. A well-known predictive indicator from earlier studies is the WHO grade. Studies showed that patients with WHO grade III meningiomas often have worse survival rates and greater recurrence rates. Therefore, many authors have recommended adjuvant RT for grade III tumors and suggested deferring RT for grossly resected grade II tumors until they recur [67,68]. 

Although surgical excision remains the gold standard therapeutic paradigm, the substantial morbidity and death rates associated with higher-grade meningiomas emphasize the need for newer and more potent treatments. There are currently some alternative adjuvant treatments available, including SRS, FSRT, interstitial brachytherapy, and fractionated EBRT. The latter group has been shown to improve LC and enhance survival, compared to observation alone, in grade III meningiomas, even when totally resected and in partially resected grade II meningiomas [69]. For individuals who cannot be operated on or are undergoing high-functional-risk surgeries, such as those for cavernous sinus meningiomas, modern RT treatment is a viable option. RT is a second-line treatment for recurrence as well as an adjuvant therapy following surgery. SRS exhibits strong LC rates with little morbidity and a comparatively high level of evidence, particularly with gamma knife devices. The FSRT results also appear to be pretty conclusive. However, there is a paucity of prospective data available and the findings are still up for debate. Indeed, when it comes to the best patient selection, scheduling of the procedure, RT equipment, volumes, or dosages, the available data are not yet conclusive. Larger tumors and/or those that are close to noble organs at risk are typically treated with FSRT. SRS delivers a single high-dose fraction over a longer period; however, it is only appropriate for highly selected patients [70]. 

For recurrent or progressive WHO grade II and III intracranial meningiomas, SRS is a helpful radical option. For this challenging population, a treatment approach emphasizing early SRS for patients with discernible tumors on postoperative imaging is expected to enhance LC and OS, and decrease radiation-related sequelae [44]. To choose the optimum set of therapeutic alternatives and ensure the best results for our patients, careful patient selection is necessary. 

Our study showed a surprisingly low number of studies on WHO high-grade meningiomas treated by SRS modalities. This small number may reflect the rarity of these tumor types and/or the more common use of conventional RT as adjuvant treatment. Many SRS series have no histologic diagnosis to show these rare histologies. The selected papers collectively presented some shared points: first, the marked worse outcome in WHO grade II relative to grade III tumors; second, the high effectiveness of SRS in tumor control even as an exclusive treatment; third, worse outcomes in recurrent cases, larger tumor sizes, and tumors with aggressive histologic markers; fourth, very limited SRS-induced complications. Therefore, SRS can be used as an alternative to surgical resection and/or postoperative conventional RT [8,19,20,71,72]. 

Some ongoing clinical trials may respond to many questions about the best management of high-grade meningiomas [73]. 

### Study Limitations 

To our knowledge, this work is the first systematic review with a meta-analysis regarding the effectiveness of SRS in the treatment of grade II and III meningiomas. The main limitation of the present review is the retrospective nature of the studies analyzed; in fact, only one prospective study was identified. The absence of prospective studies makes our conclusions less robust.

Furthermore, we decided not to include data relating to toxicity in the present work as they will be presented in a subsequent article.

## 5. Conclusions

This study emphasizes the need for more studies on the treatment of WHO grade II and III meningiomas using SRS. Current available studies, although reporting some contradictory results, show the clear benefits of SRS in the prolongation of PFS and OS. It can be used securely in primary WHO grade II meningiomas, without waiting for recurrence. SRS may be used as the sole treatment of meningiomas with promising outcomes.

## Figures and Tables

**Table 1:** Primary characteristics of included studies.

First Author	Type of Study	Number of Patients	Age	Gender	Primary/Recurrence
Hakim et al., 1998 [33]	Retrospective	G2 (*n* = 26); G3 (*n* = 18)	61.5 (median)	-	Recurrence
Ojemann et al., 2000 [34]	Retrospective	G3 (*n* = 22)	54.2	9M/12F	Primary and recurrence
Stafford et al., 2001 [35]	Prospective	G2 (*n* = 13); G3 (*n* = 9)	58 (median)	-	Primary
Harris et al., 2003 [36]	Retrospective	G2 (*n* = 18); G3 (*n* = 12)	57.8	17M/13F	Primary
Hoffmann et al., 2005 [37]	Retrospective	G2 (*n* = 15)	51	6M/9F	Primary and recurrence
Kano et al., 2007 [38]	Retrospective	G2 (*n* = 10); G3 (*n* = 2)	58.5	8M/6F	Primary and recurrence
Mattozo et al., 2007 [39]	Retrospective	G2 (*n* = 11); G3 (*n* = 1)	NR	NR	Primary and recurrence
Kondziolka et al., 2008 [40]	Retrospective	G3 (*n* = 29)	-	-	Primary
Choi et al., 2010 [41]	Retrospective	G2 (*n* = 25)	57 (median)	13M/12F	Primary
El-Khatib et al., 2011 [42]	Retrospective	G3 (*n* = 7); G2 (*n* = 9)	54	6M/10F	Primary
Kim et al., 2012 [43]	Retrospective	G2 (*n* = 25); G3 (*n* = 11)	49	16M/19F	Primary and recurrence
Pollock et al., 2012 [44]	Retrospective	G3 (*n* = 37); G2 (*n* = 13)	G2: 55 G3: 61	G2:19M/18F G3:8M/5F	Primary
Attia et al., 2012 [45]	Retrospective	G2 (*n* = 24)	NR	7M/17F	Primary
Williams et al., 2013 [46]	Retrospective	G2 (*n* = 11); G3 (*n* = 2)	48 (median)	4M/9F	Primary
Hanakita et al., 2013 [47]	Retrospective	G2 (*n* = 22)	70 (median)	11M/11F	Primary
Ferraro et al., 2014 [48]	Retrospective	G3 (*n* = 31); G2 (*n* = 4)	61	18M/17F	Primary
Bulthuis et al., 2014 [49]	Retrospective	G2 (*n* = 34)	NR	NR	Primary
Aboukais et al., 2015 [50]	Retrospective	G2 (*n* = 27)	59	9M/18F	Recurrence
Valery et al., 2016 [51]	Retrospective	G2 (*n* = 18)	68 (median)	13M/5F	Primary
Wang et al., 2016 [52]	Retrospective	G2 (*n* = 37); G3 (*n* = 9)	60.1	12M/34F	Primary
Zhang G et al., 2016 [53]	Prospective	G2 (*n* = 44); G3 (*n* = 9)	53.6	24M/29F	Primary
Kaprealian et al., 2016 [54]	Retrospective	G2 (*n* = 24); G3 (*n* = 32)	54	25M/31F	Primary and recurrence
Refaat et al., 2017 [55]	Retrospective	G2 (*n* = 75)	62	54M/43F	Primary and recurrence
Liu X et al., 2018 [56]	Retrospective	G2 (*n* = 75)	50.2 ± 14.9	33M/42F	Primary
Acker et al., 2019 [57]	Retrospective	G2 (*n* = 27); G3 (*n* = 8)	58.1 ± 15	17M/18F	Recurrence
Helis et al., 2020 [58]	Retrospective	G2 and G3 (*n* = 48)	61.226 (median)	19M/29F	Primary
Kowalchuk et al., 2021 [59]	Retrospective	G2 (*n* = 233)	60 (median)	105M/128F	Primary
Sheppard et al., 2021 [60]	Retrospective	G2 (*n* = 233); G3 (*n* = 38)	59 ± 14.1	122M/149F	Primary and recurrence
Hasegawa et al., 2021 [61]	Retrospective	G2 (*n* = 68)	NR	NR	Primary and recurrence

Abbreviations: *n*: number; M: male; F: female; G: grade; NR: not reported.

**Table 2:** The details of SRS treatment for WHO grade 2 and 3 meningiomas.

First Author	Treatment	Median Dose (Gy)	Median Fup (m)	Progression-Free Survival	Overall Survival
Hakim et al. [33] 1998	LINAC	15	31	G2: 91.7, 83.3, 83.3, and 83.3% (1, 2, 3, 4-year); G3: 92.3, 64.6, 43.1, and 21.5% (1, 2, 3, 4-year)	G2: 83%(4-year); G3: 22% (4-year)
Ojemann et al. [34]	Gamma Knife	16	29	32% (2-year); 26% (5-year)	75% (2-year); 40% (5-year)
Stafford et al. [35]	Gamma Knife	16	40	NR	G2: 76% (5-year); G3: 0% (5-year)
Harris et al. [36]	Gamma Knife	14.9	27.6	G2: 83% (5-year); G3: 72% (5-year)	G2: 59% and 59% (5 and 10-year); G3: 59% and 0% (5 and 10-year)
Hoffmann et al. [37]	Gamma Knife	16	35	NR	NR
Kano et al. [38]	LINAC	18	43.4	60%, 50%, and 50% (1, 2, and 5-year)	90%, 80%, and 80% (1, 2, and 5-year)
Mattozo et al. [39]	LINAC, Gamma Knife	15.5	42	G2: 100% (3-year); G3: 0% (1-year)	NR
Kondziolka et al. [40]	Gamma Knife	14	48	NR	22% (5-year)
Choi et al. [41]	CyberKnife	22	28	100% and 73% (2 and 3-year)	NR
El-Khatib et al. [42]	LINAC	14	60	G2: 88%, 75%, and 75% (3, 5, and 10-year) G3: 57%, 57% and 43% (3, 5, and 10-year)	NR
Kim et al. [43]	Gamma Knife	16	-	NR	NR
Pollock et al. [44]	Gamma Knife	15	38	45% (5-year)	27% (5-year)
Attia et al. [45]	Gamma Knife	14	42.5	40% and 25% (1, 2, and 5-year)	92%, 67%, and 52% (1, 2, and 5-year)
Williams et al. [46]	Gamma Knife	16	50	92%, 73%, 63%, and 31% (1, 2, 3, and 4 years)	NR
Hanakita et al. [47]	Gamma Knife	18	23.5	NR	68% (5-year)
Ferraro et al. [48]	Gamma Knife	18	34.5	G2: 70.1% (3-year); G3: 0% (3-year)	G2: 83.4% (3-year); G3: 33.3% (3-year)
Bulthuis et al. [49]	Gamma Knife	13	41	83.4% and 64.4% (2 and 5-year)	NR
Aboukais et al. [50]	Gamma Knife	15.2	56.4	NR	NR
Valery et al. [51]	Gamma Knife	15	36	G2: 71%, 36%, and 23% (1, 2, and 3-year)	NR
Wang et al. [52]	Gamma Knife	13.1	32.6	83.3%, 30.6%, 20.4% (1, 3, and 5-year) 76.2%, 25.4%, 25.4% (1, 3, and 5-year)	G2: 97.1%, 88.3% (3, 5-year); G3: 66.7%, 66.7% (3, 5-year)
Zhang M et al. [53]	CyberKnife	15	23.6	NR	G2: 97%, 87%, and 87% (1, 3, 5-year); G3: 50%, 25% (1, 2-year)
Kaprealian et al. [54]	Gamma Knife	NR	75.9	G2: 56% (5-year); G3: 47% (5-year)	NR
Refaat et al. [55]	Gamma Knife	16	41	NR	88.6% and 81.1% (3, 5-year)
Liu X et al. [56]	Gamma Knife	13	70	89.3%, 72.6%, 59.3% (1, 3, 5-year)	97.2% and 89.8% (2, 5-year)
Acker et al. [57]	CyberKnife	G2: 23.1 G3: 19.3	-	G2: 93%, 73%, and 59% (1, 3, and 5-year) G3: 93% and 46% (1 and 2-year)	NR
Helis et al. [58]	Gamma Knife	15	44	45.8% (5-year); 25.8% (8-year)	74.7% (5-year); 56% (8-year)
Kowalchuk et al. [59]	Gamma Knife	15	37.6	53.9% and 33.1% (1 and 3-year)	NR
Sheppard et al. [60]	Gamma Knife	14.8	37.8	G2: 84.2%, 67.8%, and 36.4% (1, 2, and 5-year); G3: 76.3%, 59.9%, and 20.4% (1, 2, and 5-year)	G2: 97.0%, 82.9%, and 67.2% (2, 5, 10-year) G3: 86.0%, 50.6%, and 40.5% (2, 5, 10-year)
Hasegawa et al. [61]	Gamma Knife	NR	52	52%, 35%, and 25% (3, 5, and 10-year)	85%, 78%, and 61% (3, 5, and 10-year)

Abbreviations: Gy: gray; Fup: follow-up; m: months; G: WHO grade; NR: not reported.
